# Retrospective Analysis of Standard Operating Procedures for Intraocular Lens Inventory and Stocking

**DOI:** 10.7759/cureus.84588

**Published:** 2025-05-21

**Authors:** Manda Nirmala Jyothi, Gurunadh S Velamakanni, Kandula Satish, Manne Sri Hari Babu, B V Satyanarayana

**Affiliations:** 1 Ophthalmology, Ganni Subba Lakshmi (GSL) Medical College, Rajahmundry, IND; 2 General Medicine, Ganni Subba Lakshmi (GSL) Medical College, Rajahmundry, IND

**Keywords:** age, cataract surgery, commonly used iol, intraocular lens (iol), national health service (nhs), stocking of iol

## Abstract

Aims and objectives

To analyze the most frequently used intraocular lens (IOL) powers for data-driven inventory planning and improved surgical readiness in a hospital setting.

Background

Cataract surgery requires the use of IOLs with varying powers to meet the diverse refractive needs of patients. As a result, the surgical team must be equipped with a wide range of IOL powers to ensure optimal visual outcomes. However, managing and maintaining such an extensive inventory can often be challenging and may affect the efficiency of surgical operations. This raises an important question: What constitutes an optimal and practical working IOL inventory for an ophthalmic surgical unit? Addressing this issue is essential to streamline surgical workflow while ensuring that patient needs are adequately met.

Methods

This retrospective descriptive study involved the analysis of data from 1,000 IOLs that were implanted during cataract surgeries. The primary objective was to identify the most frequently used IOL powers by reviewing the distribution and frequency of the implanted lens powers.

Results

The analysis of IOL powers implanted in 1,000 cataract surgery patients revealed a bell-shaped distribution, indicating a normal pattern of lens power usage. The IOL powers ranged from 15.0 to 31.0 diopters (D), with a mean ± SD value of 21.5 ± 1.98 D. The most frequently implanted lenses were in the 21.00-21.50 D range, accounting for 24.5% (n = 245) of all cases. In contrast, extreme IOL powers, those below 17.00 D (n = 07, 0.7%) and above 26.00 D (n = 8, 0.8%), were rarely used, highlighting their infrequency in the surgical population studied.

Conclusion

IOL powers within the range of 19.5-23.5 D, with a mean ± SD of 21.5 ± 1.98 D, were the most frequently used. Therefore, maintaining IOLs in this range, specifically 19.5, 20.0, 20.5, 21.0, 21.5, 22.0, 22.5, 23.0, and 23.5 D, would constitute a practical and evidence-based core inventory for any ophthalmic surgical unit.

## Introduction

The natural human eye consists of a crystalline lens, which allows the transmission of light into the eye. The crystalline lens plays a crucial role in focusing light, enabling us to see the world clearly. Cataract is characterized by the opacification of the lens, leading to impaired light transmission and reduced visual acuity. Over the past 30 years, the number of individuals affected by blindness and moderate to severe vision impairment (MSVI) due to cataracts has increased [1].

Cataract is the leading cause of reversible blindness in India. The prevalence of cataract increases with age, leading to an increased demand for effective surgical interventions. Globally, cataract surgery is one of the common surgical procedures [2]. The selection of an appropriate intraocular lens (IOL) is crucial for optimal visual outcomes. Accurate IOL power selection not only enhances patient satisfaction but also minimizes the need for postoperative corrective measures. In fact, cataract surgery is a refractive procedure, which means it can correct vision and eliminate the need for glasses [3].

India performs millions of cataract surgeries annually, and the increasing use of phacoemulsification with foldable IOLs has led to a steady rise in IOL demand. The figure was at 4 million in 2008 [4].

IOLs are critical components for cataract surgery, and effective stock management is essential to ensure the continuity of surgical services. Following the guidelines proposed by the VISION 2020 initiative, proper inventory control helps prevent surgical delays, reduce costs, and maintain patient confidence. Key strategies for efficient IOL management include identifying the most commonly used lens types and powers, maintaining accurate stock records, establishing minimum inventory thresholds, and planning procurement well in advance to avoid shortages. Furthermore, maintaining optimal storage conditions, organizing stock systematically, and establishing reliable supplier relationships are crucial elements. Implementing a structured stock management system not only improves patient care but also supports the broader goal of delivering accessible and affordable eye care to the community [5].

The operating hospital has to maintain an inventory of IOLs for the number of surgeries performed each day, and the number of IOLs with different IOL powers to be implanted becomes complex as the surgeries increase. What should be the inventory that an ophthalmic setup should cater to? 

This study aims to determine the most commonly used IOL powers in cataract surgery across different age groups and to explore the issue of IOL stocking in any unit.

## Materials and methods

The study was a retrospective descriptive analysis conducted in the Department of Ophthalmology at GSL Medical College, Rajahmundry, India. It analyzed the most recent 1,000 cases of cataract surgery with IOL implantation performed at our unit, concluding on March 31, 2024. The primary aim of the study was to determine the most commonly used IOL powers in cataract surgery across different age groups. Patients included in the study were between 35 and 97 years of age, of either gender, and had no prior ocular pathology other than cataract. This broad age range allowed for a diverse demographic, facilitating the evaluation of IOL use in various patient groups. Only spherical IOLs and uncomplicated cataract surgeries were considered, ensuring the consistency of the results. Patients with significant ocular comorbidities, including glaucoma, keratoconus, and retinal disorders that could influence IOL power selection, were excluded from the study. Additionally, cases involving combined surgeries were also excluded to minimize potential confounding variables. The study population was selected based on the availability of complete and reliable preoperative and postoperative medical records, ensuring both the accuracy and integrity of the data.

The data were analyzed using Microsoft Excel and SPSS software. Descriptive statistics, including mean, standard deviation, and frequency distribution, were used to summarize demographic characteristics and IOL power distribution across different age groups and genders.

## Results

A total of 1,000 records were analyzed. Among these, 462 (46.2%) were male patients and 538 (53.8%) were female patients (Table 1). The IOL power implanted in these patients ranged from 15.00 to 31.00 D. The mean IOL power implanted was 21.5 ± 1.98 D. The age of the study population ranged from 13 to 97 years (mean ± SD: 62 ± 10.3 years). IOL powers ranging from 21.00 to 21.50 D were the most frequently implanted, representing 24.5% of all cases (n = 245). This was followed by IOL powers between 20.00 and 20.50 D, implanted in 158 patients (15.8%). IOL powers <17.00 D and ≥26.00 D were implanted in seven (0.7%) and eight patients (0.8%), respectively.

**Table 1:** Distribution of intraocular lens (IOL) powers among the study participants; n (%)

Power (D)	Male, n (%)	Female, n (%)	Total, n (%)
<17.00	03 (0.64)	04 (0.74)	07 (0.7)
17.00-17.50	09 (1.94)	09 (1.67)	18 (1.8)
18.00-18.50	32 (6.9)	28 (5.2)	60 (6)
19.00-19.50	35 (7.5)	35 (6.5)	70 (7)
20.00-20.50	84 (18)	74 (13.7)	158 (15.8)
21.00-21.50	109 (23.59)	136 (25.27)	245 (24.5)
22.00-22.50	70 (15)	83 (15.4)	153 (15)
23.00-23.50	57 (12)	67 (12.4)	124 (12.4)
24.00-24.50	44 (9.5)	71 (13)	115 (11.5)
25.00-25.50	18 (3.8)	24 (4.4)	43 (4.2)
>26.00	1 (0.21)	7 (1.3)	08 (0.8)
Total	462 (46)	538 (53)	1000 (100)
Statistical analysis	Mean IOL power = 21.5 D		
	Standard deviation = 1.98		

A statistically significant gender difference of 0.28 D was observed in the mean IOL power, with females exhibiting higher values than males (21.72 and 21.44 D, respectively; p = 0.027) (Table 2).

**Table 2:** Gender-based differences in intraocular lens (IOL) power

IOL power	Gender	Mean IOL power (D) and standard deviation (mean ± SD)	Independent sample two-tailed t-test value	p-value
					Female	21.72 ± 1.99	2.214	0.027
					Male	21.44 ± 1.96		

**Figure 1:**
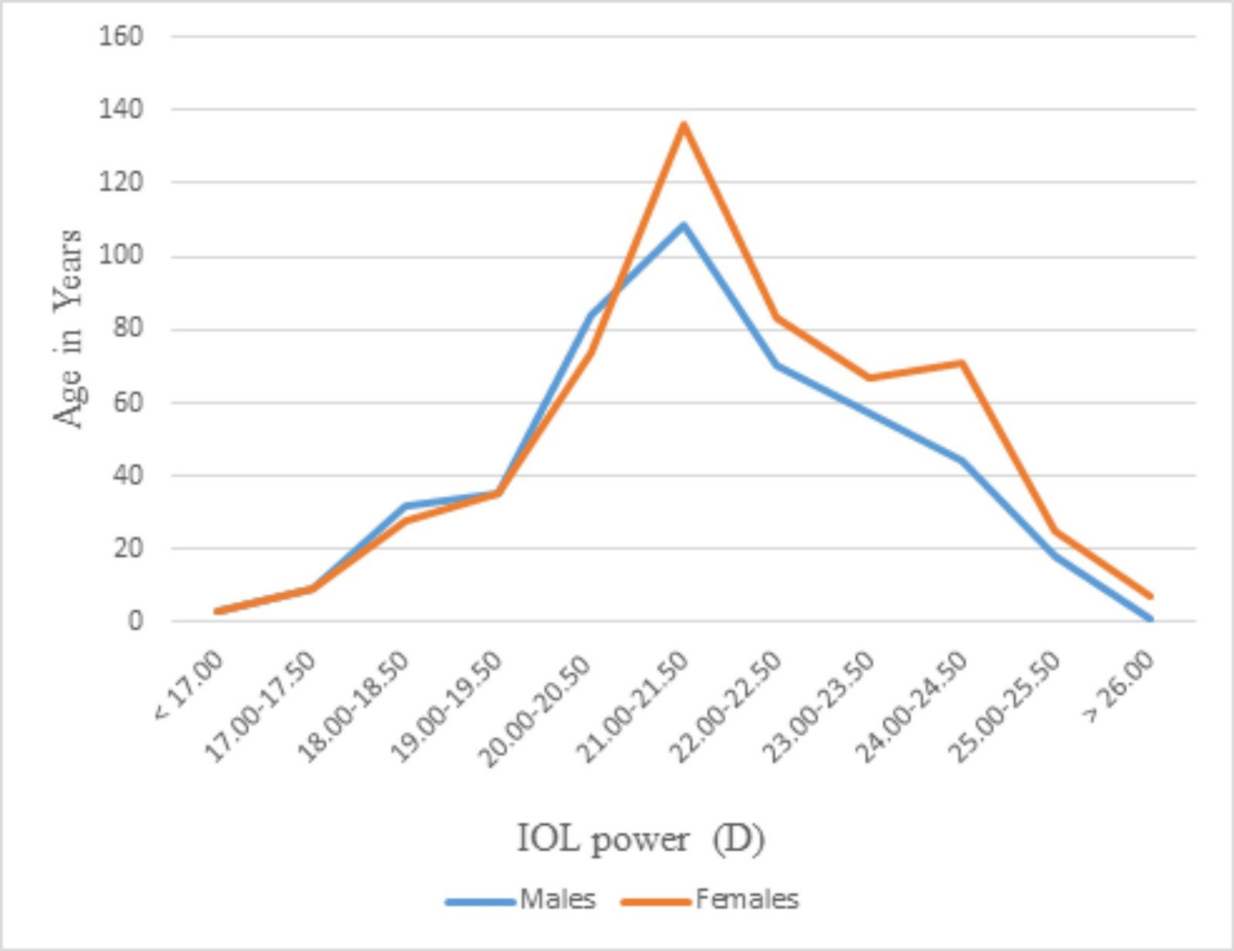
Intraocular lens (IOL) power distribution between males and females

Figure 1 shows that the IOL power distribution is not exactly bell-shaped. It is skewed toward powers between 21.0 and 24.5 D. If K is taken as 43 D and the SRK II formula is employed, the axial lengths of IOL powers from 17 to 21 D would be from 25.12 to 23.50 mm, respectively. The axial lengths for IOL powers from 21.5 to 24.5 D would be from 23.32 to 22.12 mm. This would suggest that the female population in this part of the country has shorter axial lengths.

It has been observed that more female patients had higher IOL powers compared to corresponding male patients. This would imply that female patients tend to have smaller axial lengths than male patients. The shorter axial length in females than males is in consonance with the findings in the literature [6]. 

IOL distribution among different age groups shows that the majority were in the 56-65 (n = 359, 35.8%) age group and the 66-75 (n = 318, 31.8%) age group, with mean IOL powers of 21.7 and 21.38 D, respectively. A weak negative correlation was found between IOL power and age (Pearson’s r = −0.059; p = 0.061) (Table 3).

**Table 3:** Distribution of intraocular lens (IOL) powers among different age groups

Age	Participants, n (%)	IOL power in dioptres (D)		Pearson correlation
		mean ± SD	Range	
<35	11 (1.1)	22.1 ± 1.17	18.5-31	r = -0.059, p = 0.061
36-45	52 (5.2)	21.4 ± 1.94	15-25	
46-55	192 (19.2)	21.7 ± 1.923	16-27	
56-65	359 (35.8)	21.73 ± 1.97	16-26.5	
66-75	318 (31.8)	21.38 ± 1.94	16-28.5	
76-85	58 (5.8)	21.58 ± 1.74	16-25	
86-95	07 (0.7)	21.4 ± 1.17	20.5-24.5	
>96	03 (0.3)	19.3 ± 0.92	15-22	
Total	1000			

**Figure 2:**
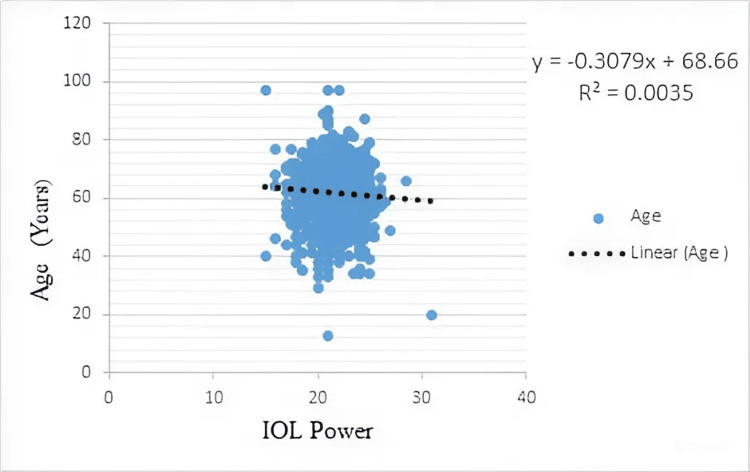
Intraocular lens (IOL) power and age

The scatter plot illustrates the relationship between IOL power and patient age. Each point represents an individual case, with IOL power on the x-axis and age on the y-axis. A linear regression line (dotted) has been fitted to assess the trend. There is a very weak negative correlation between IOL power and age, as indicated by the regression equation y = −0.3079x + 68.66 and a low coefficient of determination R² = 0.0035. It was observed that the IOL power of 23 D at 35 years of age has come down to 21 D at 86 years of age (Figure 2).

## Discussion

The main purpose of this study was to focus on the possible inventorying of IOLs in any surgical unit. A survey carried out in 10 NHS eye surgery theatres found that the system for managing and storing IOLs was not well-organized. A standardized and consistent method for monitoring lens requirements and managing inventory levels was not evident. Instead, the process appeared to be random, with lens supplies based mostly on previous use rather than any planned system. This study felt that a lack of organization could cause problems, especially in situations where less common lens powers are needed during surgery [7]

The present authors couldn’t find any published guidance on how to determine the number of lenses to retain based on the number of surgeries being performed. This was worrying, especially because hospitals were trying to work more efficiently by cutting down on waste and costs.

In most places, the number of IOLs kept in stock was based on what had been used before. They followed a simple “use and replace” method, often working with the companies that made or supplied the lenses. But this system didn’t work well if there was a problem during surgery. For example, if a one-piece lens couldn’t be placed properly in the eye and there was no suitable backup lens with the right power, the surgery could be delayed or the patient could end up with worse vision [7].

The study on the National Health Service (NHS) found that this issue became more common as hospitals tried to do more surgeries in a shorter time and run more theatre lists at once. It got even harder when weekend surgeries were added, often by private cataract providers working in NHS theatres. These changes made it even more difficult to manage equipment like IOLs properly.

Based on our survey, the recommended powers of IOLs to be kept ready in any inventory would be 21.5 ± 1.98 D (19.5, 20.0, 20.5, 21.0, 21.5, 22, 22.5, 23.0, and 23.5 D). Based on the financial resources, any unit can have multiple of these powers based on their surgery lists.

According to NICE guidelines, only one IOL matching the patient’s prescription should be in the operating theater, with at least one identical IOL kept in stock along with alternative lenses in case the selected IOL needs to be changed due to surgical complications [8]. The findings of this study support a practical adaptation of these guidelines. For routine, uncomplicated surgeries, maintaining two sets of IOLs is practical and sufficient. In complicated cases like posterior capsular rupture (PCR), a secondary IOL of the same power as the planned lens should be available, regardless of the surgical method. Effective inventory management is also essential, and unit administrators should develop strategies for recycling or reallocating lenses that approach expiry date, to avoid unnecessary wastage and ensure optimal use of resources.

This study also found that females had higher IOL powers than males. It was also observed that IOL powers declined with age. In the <35 age group, IOL power was 23.00 D (22.7 mm axial length), and for the >96 age group, it was 19.50 D (24.12 mm axial length), implying that there is an increase in the axial length of the individuals with age. This is in consonance with the works of Roy et al. and Nangia et al., where axial length was seen to correlate positively with age [9,10].

Further research is needed to focus on how often different lenses were used, the volume of surgeries, and the rate of complications. This would help build a robust system for managing IOL inventory.

Limitations

Being retrospective, the study relies on the accuracy and completeness of past surgical records. Any inconsistencies or missing data may have influenced the analysis. The study was conducted in a single hospital, which may limit the generalizability of the results to other regions or institutions with different patient demographics and surgeon preferences.

## Conclusions

This study highlights the need for a standardized and efficient approach to managing IOL stock in ophthalmic surgical units. The current “use and replace” method, based largely on historical usage and supplier input, lacks the reliability needed to support modern, high-volume surgical practices, especially when complications arise. A recommended stock range of 19.5-23.5 D should be readily available, with identical backups. Alternative options in stock to address intraoperative challenges have to be decided by the policies of the surgical unit. It is also important to plan for lens expiry and recycling to minimize waste.
